# Controversial Report Regarding Seroprevalence of Hepatitis B and C Viruses Among Hemodialysis Patients In Kerman Province, South-East Iran

**DOI:** 10.5812/hepatmon.7046

**Published:** 2013-03-13

**Authors:** Mojgan Noroozi Karimabad, Gholamhossein Hassanshahi, Mohammad Kazemi Arababadi

**Affiliations:** 1 Molecular Medicine Research Center, Rafsanjan University of Medical Sciences, Rafsanjan, IR Iran; 2 Immunology of Infectious Disease Research Center, Rafsanjan University of Medical Sciences, Rafsanjan, IR Iran

**Keywords:** Hepatitis B Virus, Hepatitis C, Epidemiology, Renal Dialysis

Dear Editor,

We read carefully the article by Zehedi et al., (1) which determined the prevalence of hepatitis B (HBV), C (HCV), and D (HDV) viruses as well as the human immunodeficiency virus (HIV) in hemodialysis (HD) patients within Kerman province, located in south-east region of Iran (1). It is now well established that the prevalence of various types of hepatitis viruses among HD patients is higher than the general population (2). According to the important role playing by viral infections in the pathogenesis of HD health status, the prevalence of the viral infections was evaluated in the HD patients by the authors. They have reported that the prevalence of HBV and HCV was moderate to low, while, HIV-Ab and HDV-Ab were both negative in all patients of Kerman province (1). The Authors of this letter believe that zahedi and co-worker`s results need to be interpreted more cautiously, because possible confounders and limitations of their study need to be considered. Zahedi et al., have evaluated the prevalence of HBV and also HCV using ELISA dependent technique and studied the HCV-RNA and HBV-DNA in the HCV-Ab and HBsAg positive patients, respectively, via Real-Time PCR. Based on the previous reports, it is obvious that a proportion of the HCV infected patients are unable to produce detectable antibody against HCV due to several reasons including humeral immunodeficiency (3), impaired Th2 system (4) as well as insufficient stimulation by innate immunity (5, 6). Additionally, the infected patients need enough time from the initiation point of the infection to produce anti-HCV antibodies (latent phase). Therefore, considerable cases of HCV infected individuals have been missed where merely ELISA technique is used to detect HCV infection. In contrast with Zahedi et al.’ results, our research team has reported already a high rate may be the cause of HCV infection in the HD patients within Kerman province of Iran (2). Interestingly, we have used RT-PCR to detect HCV-RNA in the patients, hence; it is likely that the prevalence of HCV infection may be in contrast to Zahedi et al., which is reported not following a moderate to low fashion in Kermanian HD patients. The controversy can also be considered for prevalence of HBV infection in the HD patients. We have also previously demonstrated that the prevalence of occult HBV infection (OBI), HBsAg-/HBV-DNA+ patients, was high in a population of Iranian blood donors, hence, again it seems that the prevalence of this form of disease could be elevated in the HD patients, who are among the mostly recipients of blood and its components. In parallel with our claim, several researchers reported this form of disease in the Iranian and worldwide HD patients (7-9). Due to the fact that, OBI is a form of hepatitis B, hence, evaluation of the HBsAg cannot be sufficient for reporting of HBV infection prevalence in the HD patients and the role played by OBI in the spread of infection in the HD patients may need more attentions. Finally, in order to collect more accurate and valid data regarding the real prevalence of HBV and HCV infection, we suggest re-evaluating the Kerman province HD patients using quantitative PCR technique.
